# SLC30 (ZnT) and SLC39 (ZIP) zinc transporter families: from gatekeepers of zinc homeostasis to promoters of tumorigenesis and targets for clinical therapy

**DOI:** 10.3389/fimmu.2025.1750534

**Published:** 2026-01-09

**Authors:** Yuqiao Zhou, Guangfa Huang, Mengling Liu, Minghui Zhang, Bowen Wu, Jinke Gu

**Affiliations:** 1 Guangdong Key Laboratory of Genome Instability and Human Disease Prevention, Department of Biochemistry and Molecular Biology, School of Basic Medical Sciences, Shenzhen University Medical School, Shenzhen, China; 2 Guangdong Key Laboratory for Biomedical Measurements and Ultrasound Imaging, National-Regional Key Technology Engineering Laboratory for Medical Ultrasound, School of Biomedical Engineering, Shenzhen University Medical School, Shenzhen, China

**Keywords:** clinical translation, SLC30 (ZnT), SLC39 (ZIP), therapeutic targets, tumorigenesis, zinc homeostasis, zinc transporters

## Abstract

Zinc is a trace element that plays important functions in gene expression, enzymatic activity and cellular signaling. Cellular zinc homeostasis is tightly regulated by two solute carrier families: SLC30 (ZnT, zinc transporter) and SLC39 (ZIP, zrt- and irt-like protein), which are responsible for the efflux and influx of zinc respectively. Increasing evidence demonstrates that disturbed zinc homeostasis is involved in a variety of diseases, as the altered expression of zinc transporters usually remodels the tumor microenvironment and promotes malignant development. Here, we review the structural properties, tissue localization, and physiological functions of ZnT and ZIP transporters, with emphasis on digestive systems, immune systems, neurobiological systems, endocrine systems, and other systems. We focus on their pro-tumorigenic mechanisms in different cancers, including hepatocellular carcinoma, colorectal cancer, pancreatic cancer, gastric cancer, glioma, breast cancer, prostate cancer, as well as other cancers. Overall, the ZIP family is commonly upregulated in malignancies and promotes tumor development, through the activation of signaling pathways by zinc influx. The ZnT family exhibits more complex and context-dependent functions, performing tumor suppressive and tumor promoting effects simultaneously. Zinc transporters show great potential as diagnostic biomarkers and therapeutic targets, with many members displaying prognostic relevance. Translational development is progressing, with antibody-drug conjugates (ADCs) against ZIP6 and small molecule inhibitors targeting ZIP7 and ZIP8 entering preclinical and clinical trials. Future studies should focus on full-length structure analysis of zinc transporters (particularly ZIP members), their spatiotemporal dynamics and zinc signaling in the tumor microenvironment, and their roles in therapy resistance, all of which are important for developing precise targeting of zinc homeostasis in cancer treatment.

## 1 Introduction

Zinc, the second most abundant trace element after iron, is essential for many biological processes including gene expression, enzyme function, neurotransmission, and apoptosis (1, 2). More than 10% of the human proteome is thought to interact with zinc, highlighting the importance of zinc in maintaining protein structure and function, primarily via zinc fingers (3, 4). Because of these crucial roles, cellular zinc levels must be tightly regulated, with zinc homeostasis now emerging as a factor in many pathologies, including cancer (5–7).

Zinc dyshomeostasis is a potent tumor promoter through multiple mechanisms. Dysregulation of zinc uptake, trafficking, sequestration, and export can perturb fundamental cellular activities such as cell proliferation, cell differentiation, and cell death (7). For example, zinc deficiency activates oncogenic signal transduction cascades, including MAPK/ERK and JNK pathways (8), and regulates epithelial-mesenchymal transition (EMT) via zinc-regulated transporters (9, 10). These disturbances in zinc balance drive malignant transformation by endowing cancer cells with bioenergetic and biosynthetic benefits (11).

Intracellular zinc homeostasis is maintained by the opposing activities of two solute carrier (SLC) families; the SLC30 (ZnT) family exports zinc from the cytoplasm, while the SLC39 (ZIP) family imports it. Together with metallothioneins that act as buffers for intracellular zinc, these transporters maintain zinc availability and subsequent signaling pathways (2). However, beyond this basic physiological function, accumulating evidence suggests that specific ZnT and ZIP members play a role in cancer development. Instead of passive regulators of zinc balance, these proteins are now considered to be active participants in tumorigenesis and therefore attractive therapeutic targets. Here we summarize the roles of zinc transporters in health and disease. We also discuss their emerging potential as diagnostic biomarkers and therapeutic targets for cancer.

## 2 Molecular landscape of the ZnT and ZIP families

**Figure 1:**
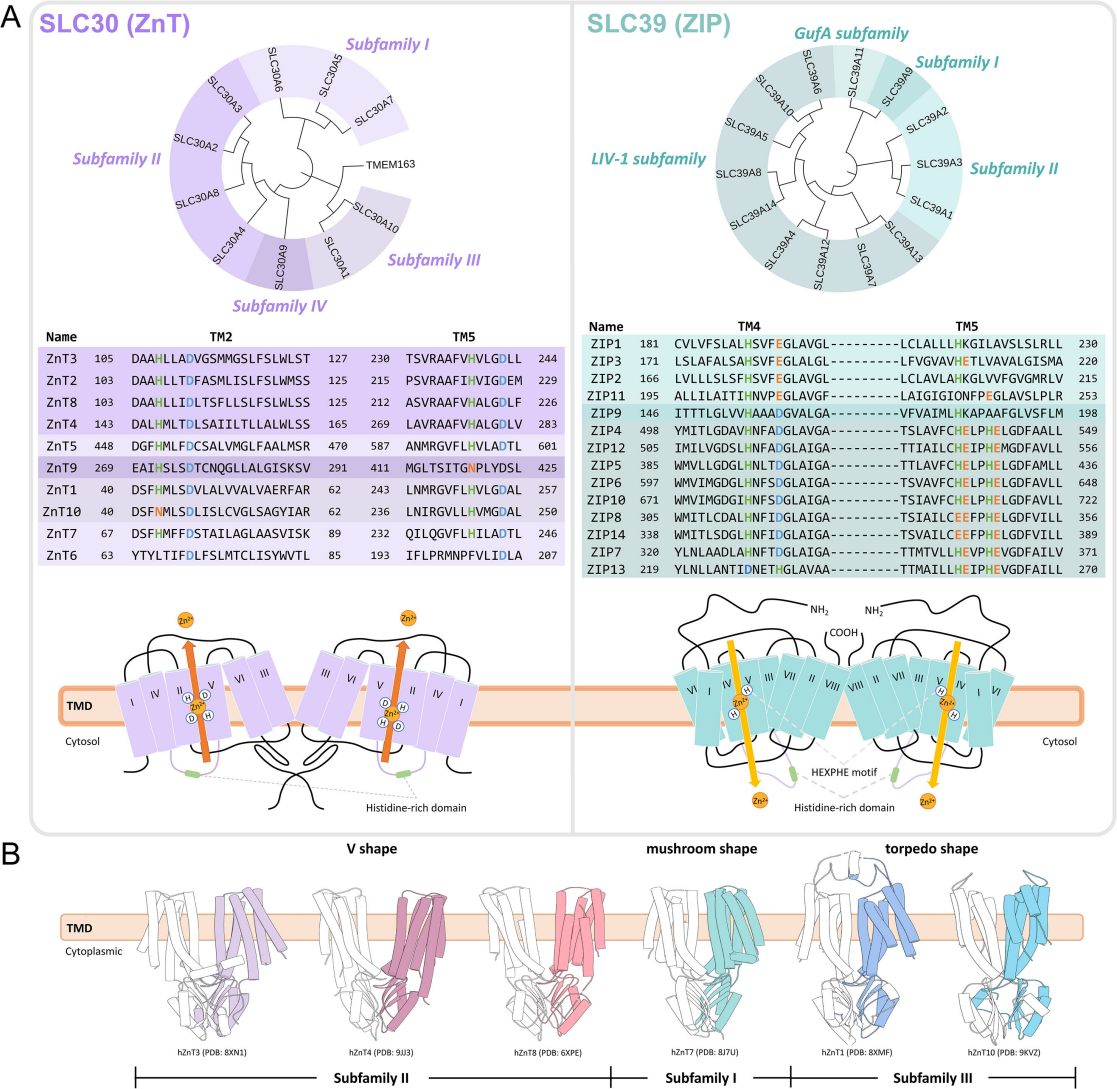
Basic information on SLC30 and SLC39 family members. **(A)** Left panel: SLC30 (ZnT) family (purple). Right panel: SLC39 (ZIP) family (cyan). The figure is organized from top to bottom as follows: Top: Phylogenetic tree. Middle: Alignment of homologous sequences at key residues (evolutionarily conserved residues are shown in color; non-conserved residues are in black). Bottom: Topology model of the zinc transporters. Orange arrows indicate Zn^2+^ efflux (from the cytoplasm to the extracellular space or organelle lumen); yellow arrows indicate Zn^2+^ influx (into the cytoplasm). **(B)** SLC30 family members exhibit distinct structural shapes across different subfamilies. ZnT3, ZnT4, and ZnT8 all belong to subfamily II and form a typical V-shaped dimer, characterized by well-separated transmembrane domains (TMDs) at its center. In contrast, ZnT7, a member of subfamily I, assembles into a tighter mushroom-shaped dimer with more extensive TMD-TMD interactions. Meanwhile, ZnT1 and ZnT10, both classified under subfamily III, associate to form compact dimers that present a torpedo-like shape.

**Figure 2:**
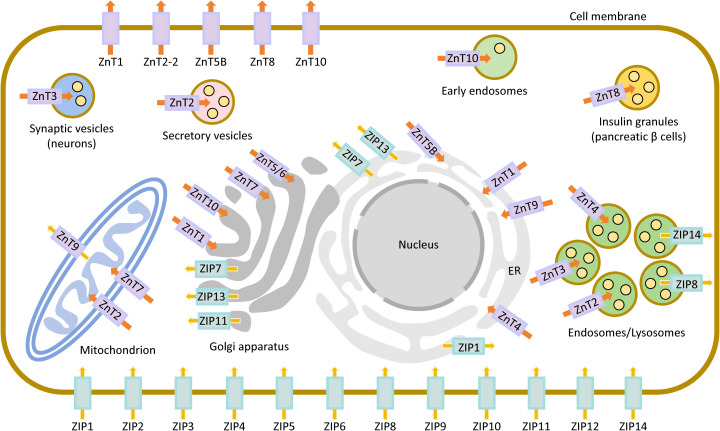
Subcellular localization of SLC30 and SLC39 family members. The SLC30 (ZnT) and SLC39 (ZIP) families are colored purple and cyan, respectively. Orange arrows show the efflux of Zn^2+^ from the cytoplasm to the extracellular space or organelle lumen, and yellow arrows show the corresponding influx into the cytoplasm.

To provide context for their physiological and pathological roles, we first outline the distinct molecular characteristics of the ZnT and ZIP families. This section covers their evolutionary classification, tissue distribution, subcellular localization, and structure-function relationships (**Figures 1**, **2**; **Table 1**).

**Table 1:** Basic information on SLC30 and SLC39 family members.

Family	Subfamily	Gene name	Protein name	Locus	Subcellular location (Simplified)	Expression (Key tissues/Conditions)	Substrate & notes
SLC30	I	*SLC30A5*	ZnT5	Chr5 (q13.1-13.2)	Iso1:Golgi apparatus/stack/TGN membrane; Cytoplasmic/secretory/COPII vesicle membrane. Iso2: ER, cell, apical membrane.	Ubiquitous. High: pancreas, liver, kidney. Beta cells (protein level).	Zn^2+^
		*SLC30A6*	ZnT6	Chr2 (p22.3)	Golgi apparatus, TGN membrane.	Brain (cerebellum, hippocampus); B-cells, colon, eye, lung. Lower in bone, cervix, etc.	Zn^2+^
		*SLC30A7*	ZnT7	Chr1 (p21.2)	Golgi/TGN/SR membrane; Cytoplasmic vesicle; Mitochondrion.	High: megakaryocytes, bone marrow, small intestine. Testis (Leydig cells), adrenal & pituitary glands.	Zn^2+^
	II	*SLC30A2*	ZnT2	Chr1 (p36.11)	Iso1: Secretory granule, endosome/lysosome, mitochondrion inner membrane. Iso2: Cell membrane.	Intestine, kidney, seminal vesicles, testis (Rat).	Zn^2+^
		*SLC30A3*	ZnT3	Chr2 (p23.3)	Synaptic vesicle membrane; Late endosome, lysosome membrane.	Brain-specific (hippocampus, cortex). Testis germ cells (Mouse).	Zn^2+^
		*SLC30A8*	ZnT8	Chr8 (q24.11)	Secretory vesicle membrane; Cell membrane.	Pancreatic beta cells. Subcutaneous fat (lean > obese). Variable in PBMCs.	Zn^2+^
		*SLC30A4*	ZnT4	Chr15 (q21.1)	Endosome, late endosome, lysosome membrane.	High: brain, testis (Rat). Also: small intestine, lung, kidney, stomach, colon (Rat). High: brain, mammary epithelium (Mouse).	Zn^2+^
	III	*SLC30A1*	ZnT1	Chr1 (q32.3)	Cell membrane (basolateral); ER, Golgi, nucleus membrane.	Ubiquitous (Human). Duodenum, jejunum, neuroglial cells (Rat).	Zn^2+^
		*SLC30A10*	ZnT10	Chr1 (q41)	Cell membrane; Golgi, recycling/early endosome membrane.	Fetal liver & brain. Adult: small intestine > liver > testes > brain > ovary > colon > cervix > prostate > placenta. Liver & neurons (protein level).	Mn^2+^/Zn^2+^
	IV	*SLC30A9*	ZnT9	Chr4 (p13)	Mitochondrion membrane; Nucleus; ER.	Ubiquitous (fetal/adult tissues, cancer lines).	Zn^2+^
SLC39	I	*SLC39A9*	ZIP9	Chr14 (q24.1)	Golgi/TGN, cell membrane; Cytoplasm (perinuclear); Mitochondrion; Nucleus.	High: pancreas, testis, pituitary. Moderate: kidney, liver, uterus, heart, prostate, brain.	Zn^2+^
	II	*SLC39A1*	ZIP1	Chr1 (q21.3)	Cell membrane; ER membrane.	Ubiquitous (adult/fetal tissues, epidermis).	Zn^2+^ (Inhibited by Ni^2+^).
		*SLC39A3*	ZIP3	Chr19 (p13.3)	Cell membrane (apical).	High: testes, hippocampus dentate gyrus (Mouse). Mammary gland (Mouse).	Zn^2+^
		*SLC39A2*	ZIP2	Chr14 (q11.2)	Cell membrane.	Prostate & uterine epithelial cells.	Zn^2+^, Cd^2+^, Cu^2+^, Co^2+^ (Active at pH 6.5; Inhibited by high K^+^).
	LIV-1	*SLC39A13*	ZIP13	Chr11 (p11.2)	Golgi, cytoplasmic vesicle, ER membrane.	High: bone, eye. Osteoblasts, odontoblasts, dermal fibroblasts (Mouse).	Zn^2+^
		*SLC39A7*	ZIP7	Chr6 (p21.32)	ER membrane; Golgi, cis-Golgi network membrane.	Ubiquitous (Human). High: intestinal crypts (Mouse).	Zn^2+^
		*SLC39A6*	ZIP6	Chr18 (q12.2)	Cell membrane (apical, lamellipodium, raft).	High: breast, prostate, placenta, kidney, pituitary. Low: heart, intestine. High in adenocarcinoma cells.	Zn^2+^
		*SLC39A10*	ZIP10	Chr2 (q32.3)	Cell membrane (apical).	Liver, kidney, brain (Mouse).	Zn^2+^
		*SLC39A5*	ZIP5	Chr12 (q13.3)	Cell membrane (basolateral).	Liver, kidney, pancreas, small intestine, colon, spleen; fetal liver/kidney.	Zn^2+^
		*SLC39A4*	ZIP4	Chr8 (q24.3)	Cell membrane (apical); recycling endosome membrane.	High: kidney, small intestine, stomach, colon, jejunum, duodenum.	Zn^2+^
		*SLC39A12*	ZIP12	Chr10 (p12.33)	Plasma membrane.	Brain, eye (Human). High: brain regions, testis germ cells (Mouse).	Zn^2+^
		*SLC39A8*	ZIP8	Chr4 (q24)	Cell membrane (apical/basolateral); Lysosome membrane.	Ubiquitous. High: pancreas. Macrophages; BBB endothelial cells (protein level).	Zn^2+^, Mn^2+^, Cd^2+^, Fe^2+^, Co^2+^
		*SLC39A14*	ZIP14	Chr8 (p21.3)	Cell membrane (apical/basolateral); Endo/lysosome membrane.	Ubiquitous. High: liver, pancreas, fetal heart. Neurons, osteoblasts, osteoclasts; BBB endothelial cells; macrophages (protein level).	Zn^2+^, Mn^2+^, Cd^2+^, Fe^2+^
	GufA	*SLC39A11*	ZIP11	Chr17 (q24.3-q25.1)	Cell membrane; Nucleus; Cytoplasm; Golgi.	High: testes, stomach, ileum, cecum. Low: liver, duodenum, jejunum, colon (Mouse).	Zn^2+^, Cu^2+^

Data sources: Locus, UCSC (https://genome.ucsc.edu/); all other fields, UniProt (https://www.uniprot.org/). TGN: trans-Golgi network, ER: Endoplasmic Reticulum, SR: Sarcoplasmic Reticulum, PBMCs: Peripheral Blood Mononuclear Cells, BBB: Blood-Brain Barrier, Iso: Isoform. (This table was compiled from the respective entries on the UCSC and UniProt websites; data are for human unless otherwise specified.).

### 2.1 The basic knowledge of ZnT

The ZnT family belongs to the cation diffusion facilitator (CDF) family, and its members act as Zn^2+^/H^+^ antiporters, exporting zinc from the cytoplasm against its electrochemical gradient, and thereby safeguarding cells from zinc overload and toxicity (2). In humans, the ten main members of the ZnT family (ZnT1-10) are highly conserved in evolution, even though their genes are located on different chromosomes (**Figure 1A**, **Table 1**).

Phylogenetic analysis of ZnT1–10 separates the ZnT family into four major subfamilies. Subfamily I includes ZnT5, ZnT6, and ZnT7, which exhibit the greatest mutual amino acid sequence identity. Subfamily II includes ZnT2, ZnT3, ZnT4, and ZnT8, which have high sequence homology and contain similar structural features such as the HCH motif. Subfamily III includes ZnT1 and ZnT10, two highly homologous transporters predominately located at the plasma membrane. Subfamily IV contains only ZnT9, which is markedly different from other ZnTs in both amino acid sequence and subcellular localization (**Figure 1A**).

Functionally, subfamily I members are primarily localized to the early secretory pathway, particularly the Golgi apparatus. There, ZnT5 and ZnT6 form a heterodimer that functions together with ZnT7 to transport zinc into this compartment. This process is essential for the activation of zinc-dependent enzymes, such as alkaline phosphatases (ALPs) (12, 13). Subfamily II members (ZnT2, ZnT3, ZnT4, and ZnT8) mainly function in the secretory pathway and are mostly found in the membranes of secretory vesicles, endosomes, and lysosomes. ZnT3 is highly and specifically expressed in synaptic vesicles of hippocampal and cerebral cortical neurons, where it concentrates vesicular zinc, serving as a marker for this pool and playing an important role in regulating neurotransmission (14). ZnT8 is almost exclusively expressed in pancreatic β-cells, where it transports zinc into insulin secretory granules, thereby promoting insulin biosynthesis; this has led to intense study of this protein in diabetes research (15). ZnT2 and ZnT4 are mainly expressed in the mammary gland (particularly during lactation), prostate, and intestine, where they facilitate zinc secretion into milk and prostatic fluid, respectively (16, 17). In subfamily III, ZnT1 and ZnT10 share a common localization to the plasma membrane but differ in their functions. ZnT1 functions as a general Zn^2+^/H^+^ antiporter, exporting zinc from the cytoplasm to the extracellular space (18). In contrast, ZnT10 mainly functions as a Ca^2+^/Mn^2+^ antiporter, mediating manganese efflux from cells (19). ZnT9 constitutes subfamily IV because of its low sequence similarity to other ZnTs. It is ubiquitously expressed and localizes mainly to the mitochondrial membrane, where it functions as a proton-coupled zinc antiporter. This zinc efflux activity is critical for maintaining zinc homeostasis, mobilizing zinc pools, and preserving mitochondrial morphology and function (20–22). In addition, TMEM163 is a newly identified mammalian ZnT, structurally related to the CDF superfamily and essential for maintaining zinc homeostasis in neuronal and vascular tissues (23–25) (**Figures 1A**, **2**; **Table 1**).

ZnTs generally assemble as dimers. Most are homodimers, although ZnT5 and ZnT6 form a functional heterodimer. Each monomer consists of two core domains: a transmembrane domain (TMD) with six helices (TM1-TM6) that mediates Zn^2+^ translocation, and a cytosolic C-terminal domain (CTD) with a unique metallochaperone-like αββαβ fold that is important for dimer stability and zinc sensing. Most ZnTs have a conserved HDHD motif within the TMD to tetrahedrally coordinate Zn^2+^ (**Figure 1A**, left). Despite this conserved topology, the overall structure of ZnTs is varied. ZnT8 and its subfamily members ZnT3 and ZnT4 have a typical V-shaped arrangement with well-separated TMDs in the center of the dimer (26–28). By comparison, ZnT7 is a tighter mushroom-shaped dimer with greater TMD-TMD interactions. ZnT1 has a unique torpedo-like shape due to a highly extended extracellular cysteine-rich region that forms a lasso stabilized by disulfide bonds (27, 29–31). ZnT10, which belongs to the same subfamily as ZnT1, also has a similar torpedo-shaped architecture, indicating that a compact dimerization mode is evolutionarily preferred. However, unlike ZnT1, ZnT10 lacks the extracellular cysteine-rich region and instead relies on alternative TMD and CTD contacts to form a compact dimer (32) (**Figure 1B**). These different shapes likely reflect their specialized functions in different cellular membranes (e.g., plasma membrane, Golgi, endoplasmic reticulum, endosomes). The similarity between ZnT1 and ZnT10 suggests that a similar structure-function relationship may be present in the same subfamily.

### 2.2 The basic knowledge of ZIP

The ZIP family consists of 14 members (ZIP1-14), whose genes are located on multiple chromosomes, and these proteins mediate zinc import into the cytoplasm from the extracellular space or the organellar lumen. In contrast to ZnTs, ZIP-mediated Zn^2+^ transport is independent of the H^+^-gradient but can be modulated by extracellular pH and membrane potential. Moreover, many ZIP transporters can also mediate the uptake of other divalent metal ions such as Fe^2+^, Mn^2+^, and Cd^2+^ (**Table 1**).

The ZIP family is phylogenetically subdivided into four subfamilies. Subfamily I includes only ZIP9. Subfamily II is an evolutionarily older and smaller group including ZIP1, ZIP2, and ZIP3. The largest and best studied group is the LIV-1 subfamily including ZIP4-8, ZIP10, and ZIP12-14. Finally, the gufA subfamily includes only ZIP11, named for its homology to the bacterial gufA gene (**Figure 1A**, right).

Subfamily I contains only ZIP9, a broadly expressed transporter with varied subcellular localization (33) (**Table 1**). In addition to its role in zinc transport, ZIP9 has been extensively studied for its non-canonical function as a membrane androgen receptor mediating rapid androgen signaling (34). This suggests that the physiological functions of ZIPs may include additional roles beyond ion transport.

Subfamily II members are mainly targeted to the plasma membrane to mediate Zn^2+^ influx from the extracellular space, and their functional diversity stems from different tissue-specific expression patterns. ZIP1 is expressed in all tissues examined, but down-regulated in prostate cancer, and serves as the major zinc importer from circulating blood plasma into prostate cells (35, 36). ZIP3 is highly expressed in hippocampal dentate gyrus granule cells and mammary alveolar cells to control zinc accumulation and mediate zinc reuptake from milk, respectively (37, 38). In contrast, ZIP2, which is evolutionarily distant from other members, transports multiple divalent cations such as Zn^2+^, Cd^2+^, Cu^2+^, and Co^2+^, with decreasing affinity (39). Its expression is highly cell type-specific and it regulates innate immune signaling through the maintenance of zinc homeostasis, as demonstrated in cardiomyocyte hypertrophy models (40).

Members of the LIV-1 subfamily have a highly conserved HEXPHE (His-Glu-X-Pro-His-Glu) motif in their extracellular domain, usually positioned between TM4 and TM5. This signature motif is involved in zinc binding and peptide bond hydrolysis. According to the genetic distance, the LIV-1 subfamily can be subdivided into four groups: (a) ZIP7 and ZIP13; (b) ZIP5, ZIP6 and ZIP10; (c) ZIP4 and ZIP12; and (d) ZIP8 and ZIP14. Unlike other members, ZIP7 and ZIP13 are localized in the Golgi and endoplasmic reticulum (ER) membranes, respectively, where they mobilize Zn^2+^ from the respective organellar lumen into the cytoplasm. Both are important for cellular homeostasis, with ZIP7 being especially important for mediating Zn^2+^-induced ferroptosis (41–43). Other members are mainly localized to the cell membrane. ZIP6 and ZIP10 form a heterodimer that mediates cellular zinc uptake. This zinc uptake induces EMT and is required for the zinc-dependent initiation of mitosis (44, 45). ZIP5 is expressed in a tissue-specific manner with predominant localization in intestine, pancreas, liver, and kidneys. At the cellular level, it transports zinc from the blood into intestinal epithelial cells, which is critical for systemic zinc homeostasis (46). ZIP4 is specifically and highly expressed on the apical membrane of duodenal and jejunal epithelial cells, where it acts as the main gatekeeper for dietary zinc absorption (47, 48). ZIP12 displays pronounced tissue-specificity with high expression in the nervous system, testis, and prostate. It is essential for vertebrate neural tube closure and neurite outgrowth and contributes to synaptic zinc in auditory cortex astrocytes. It also supports spermatogenesis by maintaining zinc homeostasis and providing oxidative stress protection (49–51). ZIP8 and ZIP14 are ubiquitously expressed. In addition to the plasma membrane, both are found on the lysosomal membrane. These transporters mediate the uptake of several divalent metal ions such as Mn^2+^, Zn^2+^, Cd^2+^, and Fe^2+^ (52–54), and are associated with inflammatory responses and metabolic diseases (55, 56).

The gufA subfamily member ZIP11 is enriched in mouse testes and certain digestive tissues, mainly residing in the plasma membrane, nuclear envelope, and Golgi apparatus (57, 58). It acts as a Mn^2+^ transporter, influences mitochondrial function, and represses mTORC1 signaling via regulation of cellular manganese, thus promoting longevity and anti-aging phenotypes (59) (**Table 1**, **Figure 2**).

Structurally, canonical ZIPs contain a TMD with eight helices (TM1-TM8), extracellular N- and C-termini, and a variable histidine-rich loop between TM3 and TM4 required for metal binding. Amphipathic TM4 and TM5 helices are thought to form a cavity through which metal ions are transported (60) (**Figure 1A**). ZIPs generally function as dimers; for example, the crystal structure of the extracellular domain of ZIP4 shows a functional dimer arranged around a PAL motif (61). While bacterial homologues such as BbZIP have been solved (62, 63), the structures of full-length human ZIPs remain unsolved and are a key target for future studies.

The distinct molecular features of ZnT and ZIP transporters, as detailed above, underpin their system-level integration. This foundation enables the precise spatiotemporal regulation of zinc across different tissues, which is critical for its physiological functions in major organ systems.

## 3 Gatekeepers of zinc homeostasis

Cellular zinc homeostasis is not a steady state maintained by individual transporters, but rather a dynamic balance regulated by a complex network of ZnT and ZIP transporters. This network allows for flexible and precise regulation, frequently through the counteracting activities of its components, to deliver zinc ions to the proper place, at the right time, and in the appropriate concentration for their biological functions. The following sections discuss the important roles of this regulatory network in the human systems (**Table 2**).

**Table 2:** Summary of ZIP and ZnT transporter functions in human physiological systems.

System	Family	Key members	Specific function	References
Digestive System	ZIP	ZIP4	The major apical zinc importer in enterocytes; Primary zinc absorption across intestinal epithelium; Mutation causes acrodermatitis enteropathica; Serves as an oncoprotein by inducing EMT, chemoresistance and cachexia in variou cancers	(47, 48, 73, 74, 75)
		ZIP5	Basolateral zinc import; contributes to zinc handling in the gut	(46)
		ZIP14	Modulates inflammatory responses in macrophages; Coordinates hepatobiliary manganese excretion; prevents neurotoxicity	(72, 84)
		ZIP10	Modulates immune responses by regulating T-cell function	(71)
		ZIP8	Genetic risk factor for Crohn’s disease via disrupted manganese homeostasis; Functions as tumor suppressor in specific contexts	(69, 70, 81)
	ZnT	ZnT1	Facilitates zinc efflux into portal circulation; Critical for systemic zinc distribution	(65, 66)
		ZnT5/ZnT6	Intracellular zinc handling and compartmentalization	(65)
		ZnT2	Buffers cytoplasmic zinc; enables TLR4 signaling, autophagy and host defense	(67, 68)
		ZnT1	Associated with poor prognosis in gastrointestinal malignancy	(76, 77)
		ZnT4	Functions as tumor suppressor in specific contexts	(80)
		ZnT10	Coordinates hepatobiliary manganese excretion; prevents neurotoxicity	(82, 83)
Immune System	ZIP	ZIP10	Drives zinc influx essential for macrophage pro-inflammatory activation; Promotes early B-cell survival through STAT3/5-dependent pathways	(86, 93)
		ZIP8	Attenuates NF-κB signaling; promotes IL-1β production and ferroptosis during sepsis; Supports Th17 differentiation via lysosomal zinc release; Polymorphisms predispose to colitis by disrupting gut barrier integrity	(69, 87, 88, 91)
		ZIP14	Contributes to hypozincemia during inflammation; Tumor-mediated upregulation causes T cell and macrophage exhaustion	(89, 96)
		ZIP6	Phosphorylated by Zap70 at TCR synapse; enables zinc influx for T-cell proliferation	(90)
		ZIP7	Deficiency disrupts ER-to-cytosol zinc transport; causes agammaglobulinemia	(92)
		ZIP9	tumor-mediated upregulation causes T cell and macrophage exhaustion	(95)
	ZnT	ZnT1	Mediates zinc efflux into phagosomes; creates toxic environment for pathogens	(85)
		ZnT8	Serves as major autoantigen in type 1 diabetes	(94)
Nervous System	ZIP	ZIP3	Mediates zinc reuptake into presynaptic terminals; Mediates zinc influx promoting neuronal death in epileptic models	(37)
		ZIP12	Drives neurite extension and neural tube closure via cytoskeletal regulation; Overexpression in schizophrenia	(49, 100)
		ZIP8	LoF variants contribute to schizophrenia pathogenesis	(102)
		ZIP14	Regulate manganese entry into brain; dysfunction causes parkinsonian disorders	(104)
	ZnT	ZnT3	Loads zinc into synaptic vesicles for activity-dependent release; Deficiency correlates with synaptic failure in Alzheimer’s disease	(97, 103)
		ZnT1	Interacts with NMDA receptor GluN2A subunit; modulates synaptic function; Upregulation protects against zinc-mediated excitotoxicity	(98, 99, 101)
		ZnT4-7	Show altered expression in amyloid plaques	(103)
Endocrine System	ZIP	ZIP6, 7	Implicated in β-cell zinc homeostasis and insulin processing	(108)
		ZIP4	Enhances glucose-stimulated insulin secretion	(109)
		ZIP5	Modulates insulin and glucagon secretion; impacts glucose tolerance	(110, 111)
		ZIP13	Negative regulator of beige adipocyte biogenesis by suppressing C/EBP-β	(117)
		ZIP3	Expression regulated by prolactin linking endocrine signaling	(119)
	ZnT	ZnT8	Transfer of zinc from the β-cell to insulin secretory granules; Facilitates insulin crystallization/storage/release; T2D risk factor; T1D autoantigen	(112–114)
		ZnT3, 5, 7	Complementary roles in insulin secretion dynamics	(105–107)
		ZnT1	Mutations cause aberrant sodium conductance driving aldosteronism	(115)
		ZnT10	LoF leads to manganese accumulation causing hypothyroidism	(116)
		ZnT2	Expression regulated by prolactin linking endocrine signaling	(118)
Musculoskeletal System	ZIP	ZIP1	Promotes osteoblast differentiation and bone formation	(120)
		ZIP13	LoF mutations impair BMP/TGF-β signaling, cause skeletal dysplasia	(121, 122)
		ZIP8	Drives osteoarthritis via MTF1-dependent MMP-13 expression; Knockdown depletes manganese; impairs myotube formation and proliferation	(125, 128)
		ZIP7	Complexes with NLRX1; regulates mitochondrial zinc and mitophagy; Essential for myogenesis; silencing suppresses myotube formation	(124, 130)
		ZIP14	Dysregulation promotes cancer cachexia-related muscle atrophy	(131, 132)
	ZnT	ZnT5	Deficiency leads to osteopenia; confirms bone mineralization role	(123)
		ZnT7	Knockout induces skeletal muscle insulin resistance	(126)
		ZnT1	Interacts with L-type calcium channels; mediates zinc influx and inhibits channel activity	(127)
		ZnT6	Variants protect against rheumatoid arthritis severity	(129)
Reproductive System	ZIP	ZIP6/ZIP10	Form heterodimer mediates meiotic maturation, which is necessary for the oocyte-to-egg transition	(133)
		ZIP12	Protects spermatogonia from oxidative stress	(51)
	ZnT	ZnT3	Modulate cytosolic and vesicular zinc levels in response to estrogen	(134)
		ZnT1	Mediates placental zinc efflux; impairment causes fetal growth restriction; Defense against cadmium-induced toxicity in testicular cells	(135)
		ZnT2, 4	Transport zinc into milk; LoF depletes milk zinc	(136, 137)
		ZnT8	Supports zinc accumulation in Leydig cells; facilitates testosterone biosynthesis	(138)
Respiratory System	ZIP	ZIP8	Promotes zinc influx; suppresses NF-κB; strengthens antibacterial defense	(139, 140)
		ZIP14	Drives ferroptosis in silica-induced fibrosis and acute lung injury	(141)
		ZIP2	Dysfunction underlies mucin overproduction in cystic fibrosis	(142)
		ZIP4, 5	Upregulated in lung cancer; activates PI3K/AKT signaling	(73, 143)
Urinary System	ZIP	ZIP8	Imports cadmium into proximal tubules; mediates nephrotoxicity	(145)
		ZIP14	Critical mediator of ferroptosis during acute kidney injury	(147)
		ZIP1	Accumulates high zinc in prostate; silencing reduces apoptosis in cancer	(36)
		ZIP4, ZIP6/LIV-1	Overexpressed in prostate cancer; promotes EMT	(149, 150)
	ZnT	ZnT9	Mutations cause renal tubulopathy with glucosuria/aminoaciduria	(146)
Circulatory System	ZIP	ZIP2	Protects against myocardial ischemia/reperfusion injury via STAT3	(151, 152)
		ZIP13	Maintains cardiac mitochondrial iron balance; downregulation aggravates I/R injury	(155, 156)
		ZIP8	Polymorphisms associate with hypertension and coronary disease	(157, 158)
		ZIP14	Inhibition reduces vascular smooth muscle ferroptosis and calcification	(160)
		ZIP10	Deletion causes embryonic hematopoietic failure	(159)
	ZnT	ZnT5	LoF causes lethal cardiomyopathy	(153)

^*^LoF, loss-of-function.

### 3.1 Digestive system

ZnT and ZIP transporters are essential for maintaining zinc homeostasis in human digestive system. Their coordinated activity is fundamental to healthy digestive function. Conversely, when this system is thrown out of balance, it can become a factor in the development of various gastrointestinal diseases.

Zinc uptake across the intestinal epithelium is mediated by ZIP4, the major apical zinc importer in enterocytes. ZIP4 expression is regulated by cellular zinc status in a feedback manner. And mutations in *SLC39A4*, the gene that encodes ZIP4, result in the zinc deficiency disorder acrodermatitis enteropathica (47, 64). Cellular zinc is then exported into the portal circulation mainly via the basolateral transporter ZnT1, which is critical for systemic zinc distribution (65, 66). In addition to these primary transporters, basolateral ZIP5 and the intracellular ZnT5/ZnT6 complex play auxiliary roles in zinc homeostasis and compartmentalization in the gut (46, 65).

Zinc transporters play important roles in shaping mucosal immunity. For example, ZnT2 expression in Paneth and colonic cells buffers cytosolic zinc to promote TLR4 signaling, autophagy, and host defense against pathogens (67, 68). Polymorphisms in *SLC39A8* are associated with Crohn’s disease possibly via mechanisms involving disrupted manganese homeostasis, glycosylation defects, and changes in the gut microbiota (69, 70). In the context of inflammatory bowel disease (IBD), ZIP10 and ZIP14 fine-tune the immune response: ZIP10 helps regulate T-cell function, while ZIP14 modulates inflammatory responses in macrophages (71, 72).

In gastrointestinal malignancy, zinc transporters are found to be altered with specific pathologic implications; ZIP4 serves as an oncoprotein in pancreatic cancer, gastric cancer, and hepatocellular carcinomas by inducing EMT, chemoresistance, and cachexia (73–75), while the expression of *SLC30A1* and *SLC39A6* is associated with poor prognosis (76–79). *SLC30A4* and *SLC39A8* act as tumor suppressors in a context-dependent manner (80, 81). ZnT10 and ZIP14 provide neuroprotection by exporting Mn^2+^ through the hepatobiliary system. The failure of this system leads to toxic Mn^2+^ accumulation, resulting in liver fibrosis and neuronal loss (82–84).

ZnT and ZIP families provide a fine-tuning regulatory network to maintain digestive health. Disruption of this network contributes to pathogenesis, creating prospects for diagnostic and therapeutic translation.

### 3.2 Immune system

ZnT and ZIP transporters serve as key regulators of the immune system by controlling zinc homeostasis. Through their management of zinc fluxes across membranes, they directly shape immune cell development, activation, and function.

In innate immunity, macrophages deploy zinc as a bactericide. Zinc efflux into phagosomes via ZnT1 renders an inhospitable milieu for intracellular pathogens (85). Zinc influx via ZIP10 is necessary for pro-inflammatory activation of macrophages, whose loss leads to impaired activation and apoptosis (86). ZIP8 not only suppresses NF-κB signaling through zinc-dependent inhibition of IκB kinase (IKK), but also promotes IL-1β production and ferroptosis in sepsis (87, 88). Additionally, ZIP14, induced by IL-6, participates in the hypozincemia observed in inflammation and infection (89).

In adaptive immunity, zinc transporters are involved in lymphocyte signaling. Upon T-cell receptor binding, ZIP6 is rapidly phosphorylated by Zap70. This zinc influx then activates the NFAT/NF-κB pathway, ultimately driving T-cell proliferation (90). In T-helper cells, ZIP8 is involved in Th17 differentiation through a mechanism of lysosomal zinc release (91). In B-cells, ZIP7 deficiency disrupts ER-to-cytosol zinc transfer, impairs B cell receptor (BCR) signaling, and causes agammaglobulinemia (92), whereas ZIP10 promotes early B-cell survival through STAT3/5-dependent pathways (93).

The importance of zinc transporters in immunopathology is evident in each disease state: ZnT8 is an autoantigen in type 1 diabetes (94), ZIP8 polymorphisms predispose to colitis by disrupting gut barrier integrity (69), and tumor-mediated upregulation of ZIP9/ZIP14 causes T cell and macrophage exhaustion (95, 96). Collectively, these works uncovered dysfunctions in zinc transporters as a major pathological mechanism.

Together, the data demonstrate that zinc transporters are the pivotal gatekeepers of immune homeostasis, and deregulation of them is a commonality among different inflammatory diseases. It is therefore of interest to focus on these transporters as potential targets for new immunomodulatory therapies.

### 3.3 Nervous system

ZnT and ZIP transporters act together to regulate neuronal zinc homeostasis, which is necessary for synapse function, neurodevelopment, and the pathology of neurodegenerative disease. In the hippocampus, the presynaptic ZnT3 loads synaptic vesicles with zinc to be released upon activation (14, 97). It has been shown that synaptically released zinc is transported back into the presynaptic terminal by transporters such as ZIP3 (37). On the postsynaptic side, ZnT1 directly interacts with the GluN2A subunit of NMDA receptor, modulating receptor function and dendritic spine morphology (98, 99). This trans-synaptic zinc signal is essential for synaptic plasticity and cognition.

During neurodevelopment, ZIP12 is involved in neurogenesis and neural tube closure by regulating cytoskeletal dynamics in a zinc-dependent manner (49, 100). In pathology, upregulated ZnT1 can serve to prevent zinc neurotoxicity, whereas intracellular accumulation of zinc induced by ZIP3 accelerates neuronal death in epilepsy (37, 101).

There is already clinical evidence that the dysregulation of zinc transporters is involved in neuropathological conditions. ZIP12 is overexpressed in schizophrenia, and loss-of-function mutations of ZIP8 cause phenotypic abnormalities by destabilizing glutamate signaling and promoting neuroinflammation (100, 102). Numerous ZnTs (ZnT1, ZnT3-ZnT7) have been found to be differentially expressed in the vicinity of amyloid plaques in Alzheimer’s disease (103). Moreover, ZIP14 functions in control of manganese uptake into the brain, and defects in this function can lead to accumulation of manganese and parkinsonian syndromes (104).

Taken together, these results indicate that the concerted action between ZnT and ZIP transporters assures zinc homeostasis in neural areas. On the other hand, their malfunction results in a variety of neurological disorders.

### 3.4 Endocrine system

ZnT and ZIP transporters are crucial for systemic and cellular zinc homeostasis, which is essential for the function of the endocrine system. By controlling zinc partitioning, these transporters directly regulate key endocrine processes, such as hormone biosynthesis, secretory granule maturation, and stimulated hormone secretion.

Pancreatic β-cells form a highly complex regulatory network. ZnT3, ZnT5 and ZnT7 have been shown to have overlapping effects on insulin secretion dynamics (105–107). Conversely, zinc uptake through ZIPs is also important, as ZIP6 and ZIP7 have been implicated in β-cell zinc homeostasis and insulin processing (108), whereas ZIP4-mediated zinc influx augments glucose-stimulated insulin secretion (109). In comparison, ZIP5 has been found to modulate the secretion of both insulin and glucagon towards systemic regulation of glucose tolerance (110, 111). And zinc is important for inducing insulin crystallization, storage, and secretion. The transfer of zinc from the β-cell to insulin secretory granules is mediated by ZnT8 (112). Variation at the *SLC30A8* locus, which encodes ZnT8, has been identified as a risk factor for type 2 diabetes (113). In type 1 diabetes, ZnT8 is a dominant autoantigen and ZnT8 autoantibodies predominate in new onset (114).

The role of zinc transporters for hormonal regulation is not confined to the pancreas. When mutated in the adrenal, ZnT1 results in abnormal sodium conductance and calcium level changes that cause overproduction of aldosterone and primary aldosteronism (115). Mutations in ZnT10 result in thyroid-specific Mn^2+^ accumulation with inhibition of thyroid peroxidase (TPO), resulting in hypothyroidism (116). In adipose biology, ZIP13 acts as a negative regulator of beige adipocyte biogenesis by inhibiting C/EBP-β and offers opportunities in obesity research (117). Moreover, the expression of transporters such as ZnT2 and ZIP3 is regulated by hormones like prolactin, linking endocrine signaling with zinc homeostasis (118, 119).

In sum, ZnT and ZIP transporters constitute an essential regulatory axis in endocrine physiology by maintaining zinc homeostasis. Mechanistically, their dysfunction is linked to endocrine and metabolic diseases, such as diabetes, aldosteronism, thyroid disease, and obesity.

### 3.5 Musculoskeletal system

ZnT and ZIP transporters sustain zinc homeostasis, which is essential for the structural and functional integrity of the musculoskeletal system.

In skeletal development, zinc transporters play critical yet distinct roles in skeletogenesis. ZIP1 promotes osteoblast differentiation and bone formation (120), whereas loss-of-function mutations in *SLC39A13* impair BMP/TGF-β signaling, leading to skeletal dysplasia and osteopenia as seen in spondylocheiro dysplastic form of the Ehlers-Danlos syndrome (121, 122). Similarly, *SLC30A5* deficiency disrupts bone mineralization and results in osteopenia in mice (123).

In skeletal muscle, specific zinc transporters are required for development and metabolism. *SLC39A7* is required for myogenesis, since its silencing prevents Akt phosphorylation and reduces myotube formation (124). Similarly, *SLC39A8* is needed in myoblasts to provide manganese necessary for SOD2 activation, which is required for myotube formation and proliferation (125). Also, *SLC30A7* has a relevant metabolic role, because its knockout causes skeletal muscle insulin resistance and alters fatty acid metabolism (126). Zinc transporters modulate excitation-contraction coupling, such as ZnT1, which interacts with L-type calcium channels to allow zinc influx for calcium signaling, but also directly inhibits channel activity by binding to the Cavβ subunit (127).

In joints, zinc transporters are involved in degenerative pathologies; ZIP8 drives osteoarthritis progression by inducing MTF1-dependent MMP-13 expression in chondrocytes (128), whereas *SLC30A6* variants are associated with decreased severity in rheumatoid arthritis (129). In intervertebral discs, ZIP7 associates with NLRX1 to control mitochondrial zinc and mitophagy, thereby delaying cellular senescence and tissue degeneration (130). Importantly, their involvement reaches systemic conditions, as illustrated by the fact that ZIP14 dysregulation induces cancer cachexia-induced muscle atrophy via zinc dyshomeostasis (131, 132).

The pivotal role of ZnT and ZIP transporters in musculoskeletal health positions them as promising targets for therapeutic intervention.

### 3.6 Reproductive system

ZnT and ZIP transporters regulate vertebrate reproduction by establishing the optimal zinc availability for gametogenesis, fertilization, and gestation.

In oogenesis, an inflammatory complex of ZIP6-ZIP10 mediates meiotic maturation by engaging a zinc signaling cascade. This cascade commands microtubule reorganization and chromatin condensation, which are necessary for the oocyte-to-egg transition. Additionally, this transition prepares the zygote by accumulating zinc store to generate the post-fertilization zinc spark (45, 133). Meanwhile, ZnT3 modulates cytosolic and vesicular zinc levels in response to estrogen (134).

After fertilization, placental zinc transport is essential for fetal growth and development. A crucial regulatory step is zinc efflux out of the placenta mediated by ZnT1. Disruption of this process results in fetal zinc insufficiency and subsequent growth restriction. One known cause of this functional disturbance is maternal cadmium exposure (135). During lactation, ZnT2 and ZnT4 are essential for secreting zinc into milk. The deletion or mutation of either transporter severely reduces milk zinc content (136, 137).

In the male reproductive system, zinc transporters are fundamental to spermatogenesis and endocrine function. ZnT8 has a key role in testosterone biosynthesis favoring zinc accumulation in the Leydig cells (138), while ZIP12 maintains the spermatogenic lineage by protecting spermatogonia from oxidative stress (51).

Taken together, ZnT and ZIP transporters regulate reproductive processes by controlling zinc homeostasis, and their dysregulation drives infertility, lactation failure, as well as other reproductive disorders.

### 3.7 Other systems

ZnT and ZIP transporters represent essential players for the control of respiration, urination, and circulation. Their dysfunction drives the pathogenesis of related diseases, from asthma and pulmonary fibrosis to nephropathy and hypertension.

#### 3.7.1 Respiratory system

Zinc transporters critically modulate pulmonary immunity and disease pathogenesis. ZIP8 also mediates the uptake of Zn^2+^ into lung epithelial cells and macrophages to suppress NF-κB-mediated immunity and promote antibacterial defense (139, 140). The induction of ferroptosis by ZIP14 contributes to tissue injury in silica-induced fibrosis and acute lung injury, and its upregulation worsens the pathology (141). On the other hand, dysfunctional ZIP2 leads to mucin overproduction and defective airway surface hydration in cystic fibrosis (142). Upregulated ZIP4 and ZIP5 in lung cancer activate PI3K/AKT signaling to promote tumor growth and chemoresistance (143, 144). Thus, ZIPs together control immune response, metal burden, and tissue integrity in lung.

#### 3.7.2 Urinary system

Zinc transporters play critical roles in renal and prostatic physiology. In the kidney, ZIP8 imports cadmium into the proximal tubules and directly contributes to nephrotoxicity (145). Mutations in ZnT9 cause a renal tubulopathy with glucosuria and aminoaciduria, suggesting a role in reabsorption (146). ZIP14 drives ferroptosis in acute kidney injury by altering iron homeostasis, while its inhibition protects against vascular calcification (147, 148). In the prostate, high zinc levels are maintained by ZIP1, and loss in adenocarcinoma reduces intracellular zinc and apoptosis, promoting tumorigenesis (36). Additionally, ZIP4 and ZIP6/LIV-1 are upregulated in prostate cancer, remodel the tumor microenvironment, and induce epithelial-mesenchymal transition (149, 150). Together, these transporters control metal disposition, cellular stress responses, and oncogenic transformation in urinary and prostatic tissues.

#### 3.7.3 Circulatory system

Zinc transporters are critical for cardiovascular integrity and hematopoiesis. In the heart, ZIP2 protects against myocardial ischemia/reperfusion injury through improved mitochondrial function in a STAT3-dependent manner (151, 152). Loss of function of ZnT5 leads to lethal cardiomyopathy, indicating its structural importance (153, 154). ZIP13 maintains cardiac mitochondrial iron homeostasis, and its suppression worsens ischemia/reperfusion injury by activating CaMKII and ferroptosis (155, 156). In the vasculature, inhibition of ZIP14 reduces vascular calcification by decreasing smooth muscle cell ferroptosis (148), whereas ZIP8 polymorphisms are associated with hypertension and coronary disease, possibly due to cadmium toxicity (157, 158). For blood cell production, ZIP10 is required for embryonic hematopoiesis (159), and its deficiency causes erythrocytosis via manganese accumulation and HIF-2α-mediated EPO overproduction (160). Overall, ZnT and ZIP transporters are key players in cardiac contractility, vascular health, erythropoiesis, and systemic zinc homeostasis.

In physiology, ZnT and ZIP transporters are custodians of zinc homeostasis; in cancer, this network is subverted, repurposing them as promoters of tumorigenesis. The following sections delineate the mechanisms underlying this pathogenic switch across cancer types.

## 4 Promoters of tumorigenesis

**Figure 3:**
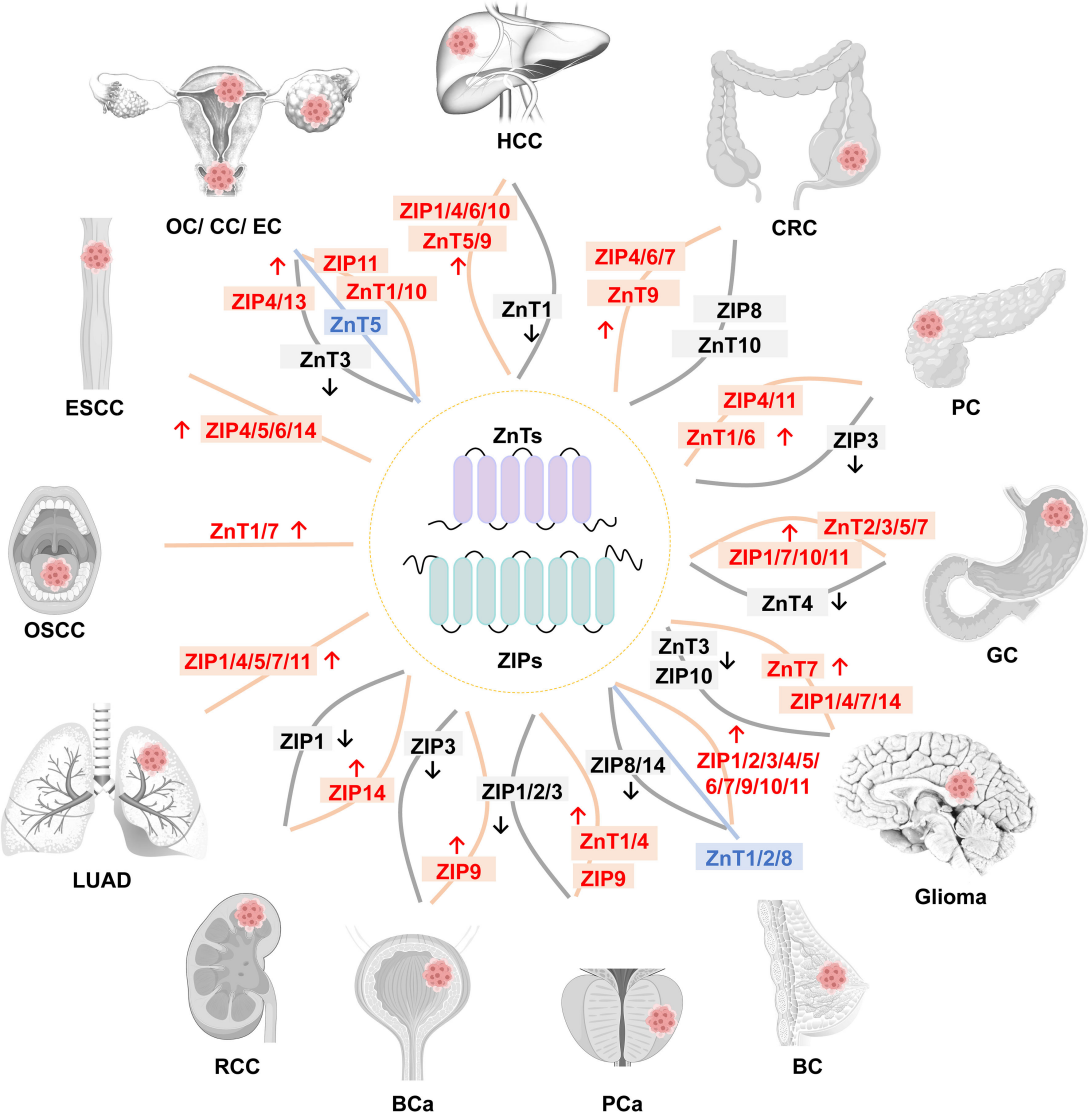
The relationship between expression of SLC30 and SLC39 family members with different cancers. Orange lines and red text represent upregulated protein in the corresponding cancer, while gray lines and black text represent downregulated protein. Blue lines and text indicate that protein expression is altered in the cancer, but the specific details are currently unknown. HCC, hepatocellular carcinoma; CRC, colorectal cancer; PC, pancreatic cancer; GC, gastric cancer; BC, breast cancer; PCa, prostate cancer; BCa, bladder cancer; RCC, renal cell carcinoma; LUAD, lung adenocarcinoma; OSCC, oral squamous cell carcinoma; ESCC, esophageal squamous cell carcinoma; OC, ovarian cancer; CC, cervical cancer; EC, endometrial cancer.

ZnT and ZIP transporters are implicated in cancer through their role in zinc homeostasis. Their dysregulation can reprogram zinc distribution and influence signaling pathways, thereby supporting tumorigenesis. While the upregulation of ZIP transporters often drives a pro-oncogenic zinc influx, ZnT activity exhibits dual, context-dependent roles. The interplay between these families constitutes a crucial regulatory layer that significantly impacts cancer progression (**Figure 3**).

### 4.1 Hepatocellular carcinoma

ZIP and ZnT transporters contribute to malignancy in HCC. Members of the ZIP family generally display pro-tumorigenic activities by mediating zinc influx. For example, ZIP1 promotes HCC progression via a DRP1-dependent mechanism that leads to mitochondrial fission and mitochondrial membrane potential (ΔΨm) loss. Its oncogenic activity is highlighted by the inhibition of proliferation, invasion, and migration after its silencing, together with reduced levels of cyclin D1, MMP2, and Wnt/β-catenin signaling components. Additionally, ZIP1 overexpression correlates with an immunosuppressive tumor microenvironment characterized by increased Th2 cell infiltration and decreased cytotoxic cell infiltration (161, 162). *SLC39A6* is also overexpressed in liver cancer and rewires mitochondrial energy metabolism by modulating the CREB1-PCK1 axis to provide energy for cancer cell motility (79). Moreover, the upregulation of ZIP4 and ZIP10 adds to this pro-oncogenic force, with ZIP10 reported to inhibit apoptosis and enhance cell migration (163, 164).

In contrast, the roles of ZnT family members in HCC are contextually complex. Rather than having a uniformly pro-tumorigenic role, ZnT1 expression is notably reduced in tumor-associated macrophages. This loss promotes inflammation and immunosuppression by impairing the zinc-dependent endosomal internalization of TLR4 and PD-L1 (165). By contrast, ZnT5 is upregulated in HCC cells and contributes to cancer hallmarks including immune infiltration, angiogenesis, and EMT (166). Moreover, ZnT9 expression is markedly increased in HCC relative to adjacent tissue and correlates with tumor progression, potentially via maintenance of mitochondrial function (167).

In HCC, ZIPs often drive tumorigenic signals, whereas ZnTs variably modulate the tumor microenvironment. This mechanistic understanding provides a rationale for combination therapies that simultaneously target multiple zinc transporters.

### 4.2 Colorectal cancer

ZIP and ZnT transporters promote CRC progression. For instance, *SLC39A4* transcription is induced by the UKLF/PCBP2 axis (168). ZIP6 is highly expressed in CRC tissues relative to normal tissue, and has been identified as a promising therapeutic target, with antibody-drug conjugate (ADC) demonstrating strong anti-tumor activity in preclinical models (169). Endoplasmic reticulum-resident ZIP7 is often overexpressed in advanced CRC, and its inhibition leads to cell cycle arrest and cell death (170). In tumor samples, decreased expression of *SLC39A8* is associated with advanced stage and poor prognosis. Mechanistically, *SLC39A8* represents a therapeutic vulnerability as it can be targeted by small molecules to induce ferroptosis (171).

ZnT family members have multifaceted and context-dependent roles in CRC. A prime example is *SLC30A10*, which has predominantly tumor suppressor activity. Its expression is often downregulated in CRC, and low levels are associated with cancer progression and metastasis; functionally, its loss promotes cancer cell proliferation and migration (172). Paradoxically, forced expression of *SLC30A10* can confer 2.7- to 4-fold resistance to the chemotherapeutic agent cisplatin in CRC (173). *SLC30A3* is dysregulated in CRC, and its increased expression results in enhanced chemosensitivity with a 3.3-fold reduction in IC50 of cisplatin (173, 174). Furthermore, ZnT9 is substantially upregulated and acts as a β-catenin coactivator to enhance Wnt/β-catenin signaling and facilitate the progression of CRC (175, 176).

This network reflects the dual clinical significance of ZIPs (like ZIP6 and ZIP8) as druggable target genes. While the context-dependent roles of ZnTs serve as prognostic markers or combinatorial strategies in CRC.

### 4.3 Pancreatic cancer

A dynamic interplay between ZIP and ZnT transporters fuels the progression of PC. The ZIP family is largely pro-tumorigenic; for instance, ZIP4 is a biomarker that is overexpressed in serum exosomes and correlates with poor prognosis (177). Mechanistically, ZIP4 promotes metastasis and EMT by upregulating the ZEB1/YAP1-ITGA3 axis and silencing ZO-1/Claudin-1 (73, 178), induces gemcitabine resistance by silencing ENT1 via ZEB1 (74), and promotes cancer cachexia by releasing vesicles via RAB27B (75). Other members of the ZIP family also have unique roles; for example, RREB1-mediated silencing of ZIP decreases intracellular zinc, thereby promoting early tumorigenesis (179), whereas ZIP11 overexpression predicts poor prognosis and promotes proliferation via ERK1/2 (77).

The ZnT family also promotes PC progression. Both ZnT1 and ZnT6 are upregulated, and the knockdown of either attenuates cancer cell proliferation by suppressing overlapping signaling pathways, including ERK1/2, p38 MAPK, and NF-κB; while ZnT1 uniquely impacts the mTOR pathway (77).

Hence, PC is dependent on a network of zinc transporters that acts in concert with ZIP4 to consolidate several hallmark features of cancer. With the help of ZnTs, this ZIP4-centric network highlights the essentiality of zinc signaling in PC and meanwhile identifies actionable biomarkers and therapeutic options.

### 4.4 Gastric cancer

In GC, ZIP family members exhibit multifaceted functions in tumorigenesis. One key mechanism involves ZIP10, which establishes a positive feedback loop with c-Myc. This loop activates zinc-dependent protein kinase CK2, thereby triggering both MAPK/ERK and PI3K/AKT signaling pathways to facilitate subsequent c-Myc expression, proliferation, and invasion (180). Therapeutically, this axis can be targeted, as exemplified by a novel STAT3 inhibitor, XYA-2, which co-suppresses *MYC* and *SLC39A10* to elicit anti-tumor effects (181). Beyond ZIP10, other ZIPs contribute to tumorigenesis: *SLC39A1* is upregulated in gastric adenocarcinoma and acts as an oncogene by promoting tumor growth and metastasis (182), whereas upregulated *SLC39A7* promotes cell growth and survival through the Akt/mTOR pathway (183). In contrast, *SLC39A11* may act as a tumor suppressor, since its higher mRNA level is associated with good prognosis (184).

In addition to the oncogenic contribution of ZIP family, the ZnT family also plays a crucial role in GC progression and prognosis. Bioinformatic analysis suggests that overexpression of ZnT family correlates with patient outcome and immune cell infiltration, and nominates *SLC30A5* and *SLC30A7* as potential prognostic biomarkers (80). Functional studies show that *SLC30A2* and *SLC30A3* play oncogenic roles (80). Experimental validation demonstrates that ZnT2 promotes proliferation, invasion, and migration, and confers resistance to zinc-induced cytotoxicity and Wnt/β-catenin signaling activation (185). By contrast, *SLC30A4* is often downregulated in GC (80).

In summary, the ZIP and ZnT families in GC provide a promising landscape for prognostic biomarkers and therapeutic interventions.

### 4.5 Glioma

*SLC30A3* is a highly expressed tumor suppressor in glioblastoma (GBM). It suppresses GBM cell proliferation and migration and induces apoptosis by activating the MAPK signaling pathway. However, its expression is silenced by the overexpressed HDAC1 in GBM tissues (186). Conversely, *SLC30A7* promotes tumorigenesis by acting as an essential cuproptosis regulator through the JAK2/STAT3/ATP7A pathway (187).

In the ZIP family, several members promote glioma progression by different mechanisms. *SLC39A1* is highly expressed in glioma tissues and associates with poor prognosis. Functionally, it promotes tumor proliferation, suppresses apoptosis, upregulates MMP2/MMP9, and may also modulate immune cell infiltration (188). Likewise, *SLC39A7* is highly expressed and correlated with poor prognosis. It promotes glioma cell proliferation, invasion, and migration via the TNF-α-mediated NF-κB pathway (189). The most elaborated mechanism is for ZIP4, which is overexpressed in GBM and associated with shorter overall survival. It promotes GBM proliferation, migration, invasion, and metastasis by upregulating TREM1 through ZEB1. Moreover, it drives the secretion of TREM1-enriched extracellular vesicles that activate microglia via the Akt-ERK1/2-STAT3 axis, thus remodeling the tumor immune microenvironment and promoting tumor progression (190). In addition, *SLC39A14* supports tumor growth and inhibits ferroptosis by activating the cGMP-PKG pathway (191). In contrast, downregulated *SLC39A10* likely acts as a tumor suppressor, as its elevated expression is correlated with improved patient survival (192).

In glioma, ZnT and ZIP transporters form an interconnected network that contributes to a pivotal role in regulating tumor growth. Although ZIPs (ZIP1, ZIP4 and ZIP7) mainly contribute to malignancy, ZnTs are functionally dualistic; ZnT3 functions as a tumor suppressor, whereas ZnT7 facilitates tumorigenesis, together orchestrating the pathogenic topology.

### 4.6 Breast cancer

ZnT and ZIP families maintain the fluency of transition between luminal and basal cells during breast cancer. Conversely, although the SLC30 family coordinates zinc efflux and trafficking, individual exporters also exhibit tumorigenic roles in cancer models, representing a highly context-dependent function.

In breast cancer, critical ZIP members promote progression by different mechanisms. The oncogene *SLC39A7* is overexpressed in the luminal A subtype, where its phosphorylation releases zinc from organelles to activate tyrosine kinase signaling pathway via receptors such as EGFR, IGF1-R, and Src, promoting proliferation, invasion, and endocrine therapy resistance (193). ZIP6 (LIV-1) has subtype-specific roles; in estrogen receptor-positive (ER^+^) cancers, STAT3 transactivates it to induce zinc-mediated EMT via GSK-3β inhibition and Snail stabilization (9), whereas its surface overexpression in triple-negative breast cancer (TNBC) predicts poor prognosis and identifies it as a target for ADCs (194). Its structurally related family member ZIP10 associates with ZIP6 to form a heterodimer that drives EMT and cancer aggression (9, 195). Several ZIP members (SLC39A1-5, 9, 11) are overexpressed in breast cancer and correlated with poor prognosis, implying oncogenicity. By contrast, SLC39A8 and SLC39A14 are more highly expressed in normal tissue, suggesting a tumor-suppressor function(196).

In parallel with the roles of ZIP transporters, ZnT family members display a complex, context-dependent regulation in breast cancer. This duality is illustrated by ZnT2; loss-of-function mutations drive malignant transformation by causing ER stress and metabolic deregulation in mammary epithelia (197), while in established tumors, ZnT2 overexpression promotes cell survival in conditions of zinc excess via vesicular sequestration (198). In contrast, ZnT1 was found to be differentially expressed in breast tumors by preferentially localizing at the plasma membrane of cancerous cells (e.g., MCF-7) but not normal epithelia, which implies a unique function for this transporter for zinc removal out of malignant cells (199). Finally, *SLC30A8* genetic variants are a significant modifier of breast cancer risk supporting their etiologic nature (200).

The ZIP and ZnT families create a co-regulated network, whose perturbation disrupts zinc homeostasis and contributes to breast-cancer progression. The ZIP family (ZIP7, ZIP6) mainly functions as tumor facilitators, whereas the ZnT family (ZnT2, ZnT1) exhibits context-dependent roles in tumorigenesis and therapy resistance.

### 4.7 Prostate cancer

PCa development is accompanied by a drastic decrease in cellular zinc due to the silencing of the zinc importers ZIP1, ZIP2 and ZIP3. These transporters act as tumor suppressors by importing zinc into cells to induce mitochondrial apoptosis. Their silencing in PCa, by epigenetic and transcriptional mechanisms, enables cancer cells to bypass this apoptotic mechanism and promotes tumor progression (201–204). In contrast to this loss of zinc import, the zinc transporter ZIP9 is overexpressed in malignant prostate tissues where its role appears to be context-dependent. When activated by testosterone or DHT, it initiates a unique apoptotic pathway. This pathway involves G protein activation followed by an increase in intracellular zinc concentration, phosphorylation of Erk1/2 and the upregulation of pro-apoptotic genes like *Bax* and *p53* (33, 34).

Accompanying the modified zinc influx, ZnT transporters that export and sequester zinc are often upregulated in prostate cancer (205). An important example is the plasma membrane transporter ZnT1 whose increased activity maintains a low cytosolic zinc concentration. Importantly, inhibition of ZnT1 or elevation of intracellular zinc results in cell death with therapeutic implications (206, 207). The role of ZnT4 seems to be context dependent. Although ZnT4 expression is higher in prostate cancer than in normal tissue from other organs, immunoreactivity for ZnT4 decreases from benign to invasive and metastatic disease (16).

Together, the ZIP and ZnT families collaboratively establish a zinc-deficient cytosol in prostate cancer—the former through lost import function (ZIP1-3) and the latter via enhanced efflux (ZnT1 and ZnT4)—collectively disabling zinc-mediated apoptosis to fuel tumor development.

### 4.8 Other cancers

In a variety of other cancers, zinc transporters have context-dependent roles in driving tumor progression. In bladder cancer (BCa), overexpressed ZIP9 acts as a membrane androgen receptor whose activation by dihydrotestosterone triggers a Gαi/MAPK/MMP9 signaling cascade promoting cell migration and invasion (208). Conversely, *SLC39A3* is downregulated, with increased expression conferring protection from BCa (209). In renal cell carcinoma (RCC), opposing roles are seen for zinc transporters; ZIP1 is a downregulated tumor suppressor that reprograms metabolism to restrain growth (210), whereas *SLC39A14* is upregulated via a circRNA-miRNA axis to promote tumorigenesis (211). Lung adenocarcinoma (LUAD) features upregulated SLC39 transporters (*SLC39A1, 4, 5, 7, 11*) associated with poor prognosis; functionally, ZIP5 activates PI3K/AKT signaling (144) and *SLC39A7* is required for cancer cell survival and growth (212). In cervical cancer (CC), *SLC39A11* displays oncogenic properties, since loss-of-function reduces malignancy (213). Meanwhile, upregulated ZnT1/ZnT10 impact the immune landscape/drug responses; ZnT1 drives broad resistance whereas ZnT10 is sensitive to specific inhibitors (Neopeltolide, Tozasertib) (214). In ovarian cancer (OC), zinc transporters ZIP4/ZIP13 promote aggressiveness via independent pathways; overexpressed ZIP4 drives drug resistance and tumorigenesis via increased expression of the stem cell marker NOTCH3 (215). Conversely, high ZIP13 levels indicate negative prognosis and its loss represses malignancy; this control is important because zinc distribution via ZIP13 changes gene profiling leading to an activation of the pro-metastatic Src/FAK (216). Altered zinc transporter expression in endometrial cancer (EC) plays a role in pathogenesis through different mechanisms. Reduced *SLC30A3* expression could disturb the intracellular zinc homeostasis and thus cause oxidative stress that contributes to all types of immune escape as well as epithelial damage. On the other hand, *SLC30A5* is related to chemotherapy resistance as its overexpression in salinomycin-treated EC cells would be a drug-triggered response to stress that allows establishing zinc metabolic homeostasis (217, 218). High levels of ZIP transporters (ZIP4, ZIP6, ZIP14, ZIP5) are associated with promoting tumor growth via several oncogenic pathways in esophageal squamous cell carcinoma (ESCC). Functionally, ZIP4 promotes growth and chemoresistance; ZIP6 enhances invasion and metastasis; ZIP14 activates PI3K/Akt/mTOR signaling; and ZIP5 supports proliferation and migration via COX2/cyclin D1 regulation (219–222). In oral squamous cell carcinoma (OSCC), distinct mechanisms involving ZnT family members contribute to pathogenesis. *SLC30A1* is transcriptionally increased in *Porphyromonas gingivalis*-infected cells and can be targeted by siRNA and zinc ionophores to induce cytotoxic zinc overload (223). Meanwhile, circRNA_100290 (a non-coding RNA associated with *SLC30A7*) exhibits remarkably increased expression levels in OSCC. It acts as a competing endogenous RNA (ceRNA) which sequesters miR-378a, thereby releasing its inhibition of GLUT1 and promoting glycolysis and cell proliferation (224).

The widespread implication of ZnT and ZIP transporters in tumorigenesis, with subtle but well-studied mechanisms between different types of cancers, also makes them an attractive therapeutic target. This has given rise to increasing efforts toward translating these findings into practical clinical applications.

## 5 Targets for clinical therapy

The families of ZnT and ZIP are central in controlling intracellular zinc levels, with their imbalance being more frequently associated with tumorigenesis, cancer evolution, and patient prognoses. This core pathogenic role highlights the outstanding value of these targets as novel therapeutic modulators in clinical oncology (**Table 3**).

**Table 3:** Clinical translational potential and druggability of zinc transporters in cancer.

Zinc transporter	Cancer type(s)	Role as a biomarker	Targeted therapeutic strategies	Clinical potential & druggability assessment	References
*SLC30A1* (ZnT1)	Hepatocellular Carcinoma, Pancreatic Cancer	mRNA or protein expression (either high or low) serves as an independent prognostic factor for poor outcomes.	Small Molecule: 3-Hydro-2,2,5,6-tetramethylpyrazine was identified as an inducer.	Primary value as a prognostic biomarker. Direct drug development is lagging, representing a fertile area for future exploration.	(76, 77, 230)
*SLC39A4* (ZIP4)	Pancreatic Cancer, Gallbladder Carcinoma, Nasopharyngeal carcinoma	A novel diagnostic and prognostic marker; high expression in tumor tissue and exosomes offers promise for non-invasive detection.	Nucleic Acid-based: Knockdown with shRNA/siRNA inhibits tumor growth and overcomes chemoresistance.	High translational potential. Supported by extensive functional genetic validation, making it highly suitable for inhibitor or nucleic acid drug development.	(10, 232, 233)
*SLC39A6* (ZIP6/LIV-1)	Breast Cancer	A powerful prognostic indicator: high levels correlate with favorable prognosis in luminal subtypes but predict poor survival and therapy failure in TNBC.	Biologics (ADC): SGN-LIV1A is in clinical trials for metastatic breast cancer BRY812 targets colorectal cancer in preclinical models	Leading translation efforts. Well-defined prognostic value and an ADC candidate already in clinical trials, establishing a successful therapeutic paradigm.	(199, 231)
*SLC39A7* (ZIP7)	Therapy-resistant Breast Cancer, T-cell Acute Lymphoblastic Leukemia	-	Small Molecule: A potent and selective inhibitor has been developed, which suppresses tumor growth by inducing endoplasmic reticulum zinc overload and stress.	High druggability. A well-characterized small-molecule inhibitor confirms its targetability, placing it close behind SLC39A6.	(227)
*SLC39A8* (ZIP8)	-	-	Small Molecule: Inhibitors have been identified via fragment-based screening and metal-chelating strategies.	Nascent potential. Emerging as a small-molecule target with identified lead compounds, but remains in early-stage discovery.	(228, 229)
*SLC39A10* (ZIP10)	Hepatocellular Carcinoma, Gastric Cancer	Elevated expression is consistently associated with unfavorable patient prognosis.	-	Nascent potential. Has broad utility as a prognostic biomarker; its role as a therapeutic target is in early exploration.	(164, 184)
*SLC39A11* (ZIP11)	Pancreatic Cancer	Elevated expression is associated with unfavorable prognosis.	-	Primary value as a prognostic biomarker. Therapeutic potential remains to be developed.	(226)
*SLC39A12* (ZIP12)	Breast Cancer, Prostate Cancer	Distinctive expression and cellular localization highlight its potential as a diagnostic marker.	Potential Biologics: Provides a rationale for developing Antibody-Drug Conjugates (ADCs).	Emerging potential. A promising diagnostic marker and therapeutic target, particularly suitable for ADC development.	(199)
*SLC39A13* (ZIP13)	Ovarian Cancer	-	Nucleic Acid-based: Knockdown significantly impedes migration and invasion of OC.	Functional validation stage. Genetic intervention demonstrates anti-metastatic effects, supporting its role as a therapeutic target.	(216)

Many of these ZnTs and ZIPs also display strong associations with patient survival across cancer types, making them promising candidates as diagnostic and prognostic biomarkers. For example, *SLC30A1* is an independent prognostic factor in hepatocellular and pancreatic carcinomas (76, 77). Likewise, ZIP4 acts as a diagnostic and prognostic marker in pancreatic cancer, with its high expression in tumors and its presence in exosomes indicating potential for non-invasive diagnosis (177). Expression of *SLC39A6* is a strong prognostic indicator in breast cancer, with high levels associated with a good prognosis in luminal subtypes, but predicting poor survival and failure to respond to treatment in triple negative breast cancer (164, 194, 225). In addition, overexpression of *SLC39A10* in hepatocellular and gastric carcinomas (164, 184) and *SLC39A11* in pancreatic cancer is consistently associated with poor prognosis (226), suggesting their widespread clinical utility.

Targeted therapeutic approaches against zinc transporters are rapidly expanding. Small molecule drugs have led to the discovery of a potent and selective inhibitor of ZIP7 that inhibits tumor growth by causing zinc overload and stress in the endoplasmic reticulum (227). Efforts to target ZIP8 have followed similar strategies of fragment-based screening and metal chelating approaches to generate leads that disrupt metal ion homeostasis (228, 229). The compound 3-Hydro-2,2,5,6-tetramethylpyrazine was identified as an inducer of ZnT1, showing potential in liver cancer models (230). Development of antibody-drug conjugates (ADCs) represents a significant step forward and several agents targeting ZIP6 show promise; ladiratuzumab vedotin (SGN-LIV1A) is in clinical trials for metastatic breast cancer (231) and BRY812 targets colorectal cancer in preclinical models (169), validating ZIP6 as a promising therapeutic target in a range of cancers. The unique expression profile of these transporters (especially *SLC39A12*) on cancer cells highlights their potential for future targeted therapeutics, such as next generation ADCs (199).

Genetic and nucleic-acid-based interventions offer a promising alternative approach; for example, RNAi mediated inhibition of the oncogenic transporter *SLC39A4* has been extensively validated in preclinical models of pancreatic, gallbladder, and nasopharyngeal carcinomas, where it suppresses tumor growth and overcomes chemoresistance (10, 232, 233). Knockdown of *SLC39A13* similarly hampers the migration and invasion of ovarian cancer cells (216).

The clinical translation of zinc transporters has a mixed but hopeful prognosis. In the short term, ZIP6 is ahead with an ADC candidate, followed by ZIP7 and ZIP8 with robust preclinical validation. The long-term potential includes the emerging ZIP targets and the underexplored ZnT family. Further advances will require structural knowledge and delivery technologies, which may cement these families of proteins as the foundation for new cancer treatments.

## 6 Conclusion and outlook

Zinc homeostasis, which is mainly controlled by the ZnT and ZIP families of transporters, is crucial for various physiological processes in various organ systems. In this review, we have explored the molecular architecture, tissue distribution, and systemic roles of these transporters, emphasizing their functions as critical regulators across multiple physiological systems, including digestion, immunity, neurology, endocrinology, and reproduction, as well as other systems. Besides having these physiological functions, there is now overwhelming evidence to support a role for these transporters in tumorigenesis. A commonality is that ZIP family proteins exhibit pivotal oncogene promiscuity due to activation of pro-growth, invasion, and survival signaling pathways. By contrast, the ZnT family exhibits a more complex, context-dependent function to fine tune malignant phenotypes towards inhibitory or promoting effects.

Dysregulated zinc transporters are associated with the progression and therapeutic resistance in several cancers, suggesting that they would be potential cancer biomarkers. Their clinical trial potential is rapidly being realized with several strategies. For instance, ADC Ladiratuzumab vedotin targeting ZIP6 shows clinical efficacy (231) and novel ADC BRY812 targeting ZIP6 reduces growth, migration, and stemness of colorectal cancer cells in preclinical studies (169). Meanwhile, advances in small molecule inhibitors of ZIP7 and ZIP8 point toward a growing target space for this protein class (227–229).

In the future, a number of important lines need to be followed up. First, the advances in structural biology to solve full-length structures of human zinc transporters, especially ZIPs, will enable rational design of specific inhibitors. Second, elucidating the spatial and temporal features of zinc signaling in the tumor microenvironment is essential to understanding how it modifies therapeutic resistance. Third, combinatorial approaches targeting oncogenic ZIP influx and compensatory ZnT-mediated efflux could have synergistic effects. Finally, the search for unconventional functions of transporters (e.g., transmembrane androgen receptor function in the context of ZIP9) may lead to the identification of novel signaling modalities and potential new therapeutic targets.

In conclusion, ZnT and ZIP zinc transporter families have been identified as a class of regulators of oncogenesis and appealing molecular targets for therapy. Their journey from bench to bedside is currently under way with the support of biomarker approaches and a broad choice of therapeutic modalities. With high intellectual and scientific challenges, future directions will be to obtain more mechanistic insights and novel targeted approaches which could take the maximum advantage of this family of proteins as anticancer drugs.
